# Supplementary material for: SARS-CoV-2 Vaccines: The Advantage of Mucosal Vaccine Delivery and Local Immunity
Source: Vaccines (Basel). 2024 Jul 18;12(7):795. doi: 10.3390/vaccines12070795 (PMC11281395; doi:10.3390/vaccines12070795)
Supplement: Supplementary file 1 [file vaccines-12-00795-s001.zip › vaccines-3079442_Supplementary Materials and Methods; 2024-07-10.pdf]

vaccines MDPI

Review

SARS-CoV-2 vaccines: the advantage of mucosal vaccine delivery and local immunity

Joshua Tobias[1*], Peter Steinberger[2], Joy Wilkinson[1], Gloria Klais[1], Michael Kundi[3], Ursula Wiedermann[1*]

[1] Institute of Specific Prophylaxis and Tropical Medicine, Center for Pathophysiology, Infectiology and Immunology, Medical University of Vienna, Vienna, Austria.
[2] Division of Immune Receptors and T Cell Activation, Institute of Immunology, Center for Pathophysiology, Infectiology and Immunology, Medical University of Vienna, Vienna, Austria
[3] Department of Environmental Health, Center for Public Health, Medical University of Vienna, 1090 Vienna, Austria
* Correspondence: Dr. Joshua Tobias, PhD (joshua.tobias@meduniwien.ac.at), and Univ. Prof. Dr. Ursula Wiedermann, MD, PhD (ursula.wiedermann@meduniwien.ac.at); Institute of Specific Prophylaxis and Tropical Medicine, Center for Pathophysiology, Infectiology and Immunology, Medical University of Vienna, Kinderspitalgasse 15, 1090 Vienna, Austria; Tel: +43-1-4016038261

Supplementary Materials and Methods

1. Peptide preparation for immunization

Synthesis and conjugation

The mimotope P#37 (KVGGNYNYLYRLFRK, AA 444-458; Accession Nr yp_009724390.1) was synthesized and conjugated to CRM197 (CRM) at piCHEM (Graz, Austria).

Mixing with the adjuvant Montanide

Montanide ISA-51-VG (Seppic, France), which is a water-in-oil emulsion, was used for mice immunization with JTMP. The calculation of the required amount of the adjuvant for the immunizations and its mixing with the conjugated peptide was carried out as previously described [1].

2. Immunization experiment

The animal experiment was approved by the Animal Experimentation Committee of the Medical University of Vienna and the University of Veterinary Medicine as well as by the Austrian Federal Ministry of Science and Research (BM:WFW-GZ 66.009/2020-0.253.778). Female BALB/c mice (n=18), aged 6–8 weeks at the time of delivery, were purchased from Charles River (Germany), kept under conventional housing conditions. The mice were divided into 3 groups (n=6). In group A, the mice received three intranasal administration of P#37-CRM197 (30µl, in both nostrils, containing 10µg of the peptide) in two weeks interval; In group B, the mice were subcutaneously injected twice with P#37-CRM197-Montanide (containing 10µg of the peptide) in two weeks interval; in group C, the mice were primed subcutaneously injected twice with P#37-CRM197-Montanide and two weeks later, boosted with three intranasal administration of P#37-CRM197, as carried out in groups A and B.

3. Evaluation of humoral and cellular responses in the immunized mice

Humoral responses

At the time of sacrifice, mice sera and bronchoalveolar lavage (BAL) fluids were collected and the level of peptide-specific IgA, IgG (total, IgG1, and IgG2a), were measured by ELISA. Microtiter plates (Nunc MaxiSorp, Denmark) were coated with P#37, in PBS (0.5 μg/well), and ELISA was performed as previously described [1,2]. After blocking, diluted sera or BAL fluids were added into the coated wells. The examined bound antibodies were detected as follows: IgA: rat anti-mouse IgA (BD eBioscience, #556960) followed mouse anti-rat IgG-HRP (Jackson ImmunoResearch, #212035168); IgG: rabbit anti-mouse IgG-HRP (Jackson ImmunoResearch, #315035008); IgG1: rat anti-mouse IgG1-HRP (BD eBioscience, #553440); IgG2a: rat anti-mouse IgG2a-HRP (BD eBioscience, #553387). The ELISAs were developed by TMB staining, stopped with H2SO4 solution, and plates were read at 450 vs 630 nm.

Cellular responses (Cytokines production in cultures of splenocytes)
The sacrificed mice splenocytes were taken aseptically, minced, sterile-filtered and cell suspensions were prepared as previously described [20]. Cells (5x105 per well) were plated in 96-well round-bottomed plates, and stimulated with unconjugated P#37 at concentration of 40μg/ml for 72 h in culture medium (RPMI 1640, with 10% heat- inactivated FCS, 2mM L-Glutamine) at 37°C, 95% humidity and 5% CO2. Supernatants were harvested and stored at -20°C, until analysis. Levels of secreted IL-2, IL-4 and IFN were measured by ELISA according to manufacturer's instructions (Affymetrix eBioscience, USA), and expressed in pg/ml.

4. Epitope mapping of anti-SARS-CoV-2 therapeutic monoclonal antibodies
Lyophilised biotinylated 15-mer overlapping peptides (JPT Peptide Technologies), spanning the entire RBD of ancestral SARS-CoV-2 RBD, were solubilised in 100μl 100% Dimethyl Sulphoxide (DMSO) and then diluted 1:250 in dilution buffer (PBS-0,5% Bovine Serum Albumin-0,05% Tween20) and incubated for approximately 4 hours at 4°C. Streptavidin-coated 96 well Plates (Thermo Fisher) were washed with 300μl three times to remove the BSA blocking. Each examined diluted overlapping peptides, or 15-mer unrelated peptide as negative control, were added to the Streptavidin-coated wells. The plate was sealed and left overnight at 4°C. The following day, the coated plate was washed, and the examined mAbs were diluted to 5μg/ml in dilution buffer. The plate was incubated for 2 hours at room temperature. After washing the plate, mouse anti-Human IgG Fc-HRP (Aviva System Technologies, #OASB00876) as the secondary antibody was added to the wells, and the plate was incubated for an additional 2 hours at room temperature. The ELISAs were developed by TMB staining, stopped with H2SO4 solution, and plates were read at 450 vs 630 nm.

References

1. Tobias, J.; Jasinska, J.; Baier, K.; Kundi, M.; Ede, N.; Zielinski, C.; Wiedermann, U. Enhanced and long term immunogenicity of a Her-2/neu multi-epitope vaccine conjugated to the carrier CRM197 in conjunction with the adjuvant Montanide. BMC Cancer 2017, 17, 118, doi:10.1186/s12885-017-3098-7.

2. Tobias, J.; Battin, C.; De Sousa Linhares, A.; Lebens, M.; Baier, K.; Ambroz, K.; Drinic, M.; Hogler, S.; Inic-Kanada, A.; Garner-Spitzer, E.; et al. A New Strategy Toward B Cell-Based Cancer Vaccines by Active Immunization With Mimotopes of Immune Checkpoint Inhibitors. Front Immunol 2020, 11, 895, doi:10.3389/fimmu.2020.00895.

Disclaimer/Publisher's Note: The statements, opinions and data contained in all publications are solely those of the individual author(s) and contributor(s) and not of MDPI and/or the editor(s). MDPI and/or the editor(s) disclaim responsibility for any injury to people or property resulting from any ideas, methods, instructions or products referred to in the content.
