# Supplementary figures and images for: SARS-CoV-2 Vaccines: The Advantage of Mucosal Vaccine Delivery and Local Immunity
Source: Vaccines (Basel). 2024 Jul 18;12(7):795. doi: 10.3390/vaccines12070795 (PMC11281395; doi:10.3390/vaccines12070795)

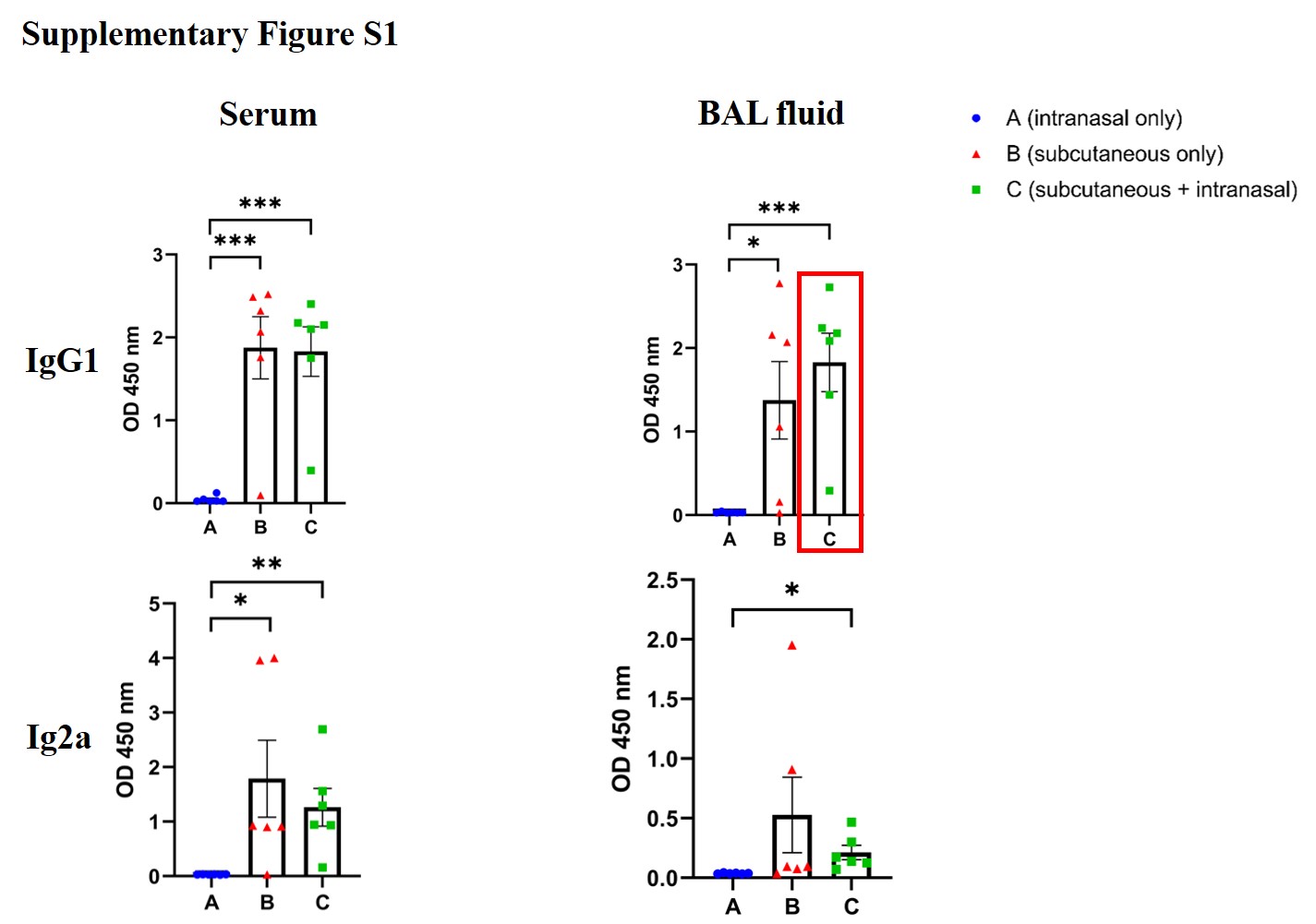

Supplement: Supplementary file 1 [file vaccines-12-00795-s001.zip › vaccines-3079442_Supplementary Figure S1; 2024-07-10.jpg]
